# DNA-informed breeding of rosaceous crops: promises, progress and prospects

**DOI:** 10.1038/hortres.2017.6

**Published:** 2017-03-15

**Authors:** Cameron P Peace

**Affiliations:** 1 Department of Horticulture, Washington State University, PO Box 646414, Pullman, WA 99164-6414, USA

## Abstract

Crops of the Rosaceae family provide valuable contributions to rural economies and human health and enjoyment. Sustained solutions to production challenges and market demands can be met with genetically improved new cultivars. Traditional rosaceous crop breeding is expensive and time-consuming and would benefit from improvements in efficiency and accuracy. Use of DNA information is becoming conventional in rosaceous crop breeding, contributing to many decisions and operations, but only after past decades of solved challenges and generation of sufficient resources. Successes in deployment of DNA-based knowledge and tools have arisen when the ‘chasm’ between genomics discoveries and practical application is bridged systematically. Key steps are establishing breeder desire for use of DNA information, adapting tools to local breeding utility, identifying efficient application schemes, accessing effective services in DNA-based diagnostics and gaining experience in integrating DNA information into breeding operations and decisions. DNA-informed germplasm characterization for revealing identity and relatedness has benefitted many programs and provides a compelling entry point to reaping benefits of genomics research. DNA-informed germplasm evaluation for predicting trait performance has enabled effective reallocation of breeding resources when applied in pioneering programs. DNA-based diagnostics is now expanding from specific loci to genome-wide considerations. Realizing the full potential of this expansion will require improved accuracy of predictions, multi-trait DNA profiling capabilities, streamlined breeding information management systems, strategies that overcome plant-based features that limit breeding progress and widespread training of current and future breeding personnel and allied scientists.

## THIS REVIEW

Fundamental principles underlie rapid advancements in use of DNA-base information to support genetic improvement of rosaceous crops and crops with similar features. No longer just promise, molecular genetics is transforming rosaceous crop breeding worldwide. However, where successful, new technologies have integrated into and certainly not replaced a core backbone of traditional breeding. Plants must still be created, raised and evaluated, within the constraints of each crop’s biological features. The production industry and consumers must still be convinced of each new cultivar’s worth to achieve intended commercial success. And breeding must be grounded in the scientific discipline of genetics—that is, the study of inheritance and its repercussions, not the study of DNA. Some reviews in this field have been published in recent years, for example, for various rosaceous crops,^1^ apple,^2,3^ strawberry,^4^ *Prunus*,^5,6^ Rosoideae^7^ and Rosaceae in general.^8^ Such reviews have commonly detailed the tools useful for genetics research (for example, marker types and platforms, genetic mapping, quantitative trait locus (QTL) characterization and bioinformatics), sometimes straying into physiology (for example, transcriptomics, proteomics and metabolomics), but have rarely focused on the applications of genomics-age tools and knowledge for genetic improvement in breeding practice. This review focuses on promises, progress and prospects of using DNA-based information for practical breeding across Rosaceae crops in general. The concepts described should also hold relevance for other horticultural crops, especially those clonally propagated and perennial.

## ROSACEOUS CROP BREEDING

### The need to breed

Rosaceous crop industries and consumers demand new cultivars. The Rosaceae family of crops (including apple, almond, apricot, blackberry, peach, pear, plum, raspberry, rose, strawberry, sweet cherry and tart cherry) provides fresh and processed products that enhance human health and well-being.^9^ The multi-billion dollar Rosaceae crop production and processing industries represent the economic backbone for many rural communities. Industries must meet increasing and dynamic marketplace needs to consistently deliver products with acceptable quality, safety and affordability while simultaneously confronting production and handling threats.^10^ These industries therefore require superior new cultivars, a demand within each crop that is driven by industry size, enhanced by genotype×environment interaction (G×E), focused on particular traits and moderated by cultivar name recognition. Significant G×E, that is, the differential relative performance of cultivars across regions, increases the need—and breeding opportunity—for scion and rootstock cultivars specifically suited to regional commercial production conditions. Determination of trait priorities by Rosaceae crop breeders, that is, differential allocation of breeding resources among traits toward their genetic improvement, involves weighing many factors, which in the US at least is positively influenced primarily by consumer-driven forces (consumers, marketers and retailers).^11^ Cultivar name recognition in the production and handling industry and at the marketplace imparts inertia that reduces prospects for additional new cultivars to enter^12,13^ but creates an opportunity for branding.

### Components of breeding

Rosaceae crop breeding is the development and delivery of genetically superior cultivars to address needs in the crop production-societal system. Available germplasm is accessed for the superior alleles therein and those individuals with genetic potential for desired performance levels are selected, thereby providing genetic improvements that raise the bar with each successive generation of new cultivar release. Breeding germplasm of rosaceous crops consists of parents (often cultivars and selections, sometimes with partially wild ancestry usually from dedicated efforts to introgress valuable wild alleles), families of offspring also called ‘seedlings’ (usually present singly), increasingly ‘elite’ selections (replicated in field trials) and commercially released cultivars.

Breeding of these crops can be considered to have four workflow stages which approximately align with the germplasm levels. Goal-setting, especially to determine what sets of attributes to target, is prominent prior to crossing, but revisited often as observations of breeding activity outcomes are considered for their effectiveness. Obtaining and creating new genetic variation is usually by controlled crossing, involving sexual reproduction, between pairs of parents which combines new sets of parental alleles into single individuals. In the selection stage, individuals determined to have the best genetic potential for a long list of traits are chosen and examined with increasing scrutiny. The chronological order and manner in which traits are evaluated depend largely on heritabilities, frequencies of desired phenotypes, plant developmental phases at which traits are expressed and resources needed for evaluations. Selection ‘for’ individuals comes with clonal propagation (selection against means discarding—usually terminal). Commercialization of new cultivars involves proving they are distinct and new, clonal propagation to large numbers suitable to commercial production and convincing growers to buy and plant them. Asexual reproduction through the last two stages captures additive, dominance and epistatic genetic action of the many alleles contributing to superior performance.^14^ Rosaceae breeding is conducted either *ad hoc* or in breeding programs, which can be public institutions or private enterprises. Numerous scientific disciplines are connected to breeding programs, but the primary underlying discipline is genetics.

### Traditional breeding approaches

Traditional rosaceous crop breeding continues to meet some demands, but is expensive and time-consuming and would benefit from improved efficiency and accuracy. Although some rosaceous cultivars with substantial market share were originally chance seedlings^13^ such as ‘Golden Delicious’, ‘Red Delicious’ and ‘Braeburn’ for apple,^15^ many were derived from planned, intentional breeding. Rosaceae breeding program traditionally rely on two forms of genetic information, phenotype and pedigree, to indicate genetic potential for superior performance under commercial conditions. Improved methods that reveal genetic potential accurately and/or efficiently would have a huge positive impact on Rosaceae breeding by reducing costs and leading to superior new cultivars released more frequently or with even greater genetic potential than from traditional methods alone.

The first traditional genetic information source, performance evaluation data, is the mainstay, because it can be readily obtained for many traits that must be considered in breeding and particularly because it is ultimately what will also be learned and experienced by growers, handlers, transporters, retailers and consumers. However, phenotype is an indirect indicator of each trait’s genetic potential except where heritability is high. For many traits of breeding interest, observing performance for many years, growing locations and management conditions is required to reveal genetic potential accurately. Shortcuts in phenotyping, such as subjective measures and few years/locations/conditions of observation, can easily lead to erroneous conclusions of genetic potential. Robust phenotyping is therefore a major concern for rosaceous crop breeding programs. Phenotyping protocols can be standardized within programs as well as across programs to raise statistical power,^16^ as conducted for apple,^17,18^ peach,^19^ strawberry,^20^ sweet cherry^21^ and tart cherry,^22^ and sophisticated statistical models can be used to account for probable confounding factors;^23–26^ but limitations remain.

The second traditional form of genetic information, parentage and ancestry, is an even more indirect measure of genetic potential, as it infers that predicted or observed attributes of breeding material are inherited from parents. Although indirect, pedigree is a deep consideration—it underlies the reason that controlled crosses (combinations between chosen pairs of parents) are a key breeding operation. The importance of pedigree is also the reason that the use of open-pollination or chance seedlings might be considered unscientific, with only one or no parents chosen as contributors to that next generation. Lack of parent selection would greatly reduce a breeder’s accuracy to predict the performance of such offspring and reduce their efficiency of intentionally combining desired attributes into single individuals. Even in the majority of breeding situations where both parents are chosen, parentage records are sometimes wrong.^27^

### Biology limits breeding effectiveness

Plant-based features strongly influence the ability of breeding to supply new rosaceous cultivars that meet industry and consumer demands. Many of the features that reduce breeding effectiveness in the context of particular breeding stages are listed here.

#### Goal setting

- Traits types (subjectivity of horticultural quality traits involving consumer preference, many traits of commercial relevance that vary in breeding germplasm, rarity of desirable trait levels in breeding families, perenniality of plants adding the dimension of time to the list of traits to consider).
- Genetic architecture of selection-targeted traits (many influencing trait loci, small allele effects).

#### New genetic variation

- Existence of and physical accessibility to desirable genetic diversity (small primary/secondary/tertiary gene pools, germplasm difficult to access because of location and/or quarantine).
- Genetic accessibility to useful alleles (small primary gene pool, polyploidy effects on reducing effective meiosis and complicating genetic architecture by increasing number and interactions of alleles, large degree of domestication leading to large genetic differences between modern cultivars and the wider allele pool such that many generations are expected to be required to incorporate useful alleles, long juvenility period and large plant size rendering introgression an enormous task).
- Ease of obtaining sufficiently sized cultivar-generating families by controlled crossing among elite parents (long juvenility period, specific environmental requirements for flowering, specific timing of stigma receptivity, asynchronous flowering of parents, short pollen viability, susceptibility to environmental hazards at flowering time such as freezes, self-compatibility that complicates intended crossing to a different parent, self-incompatibility that prevents desired selfing, few alleles for cross-compatibility in a narrow gene pool, complex arrangement or small size of floral organs leading to high labor needs for crossing, few viable seeds per crossing attempt, specific environmental requirements for germination, low germination rate of seeds, low heterozygosity leading to little diversity in families, high heterozygosity complicating predictions of outcomes in families).

#### Selection

- Efficiency of phenotype-based selection (long juvenility period, large plant size, low heritability and significant G×E of target traits, complicated genetic architecture of target traits).

#### Commercialization

- Cultivar plant propagation (not clonally propagated, if clonal: difficulty in rooting runners/cuttings or graft incompatibility, if grafts: rootstock×scion effects)

The above breeding-limiting features are commonly encountered across rosaceous crops.^13,15,28–31^ Some of these features are described in more detail below to exemplify the breeding challenges. Methods and technologies intended to support breeding effectiveness are typically those aimed at circumventing one or more of these biological constraints. Yet, innovations need to be mindful of and operate within all the other remaining constraints for that crop.

### Some biological considerations in goal-setting

Planning breeding germplasm and operations to address fruit, nut and flower quality is complex as such traits are often highly subjective especially targeting the fresh market, yet such traits are most highly prioritized by Rosaceae crop breeders within their limited resources.^10^ Sensory and other quality aspects in addition to productivity and biotic and abiotic stress resistance traits total >50 traits per Rosaceae crop.^10^ Many loci and alleles underlie these traits.

### Some biological considerations in creating new genetic variation

Rosaceae crops tend to have diverse gene pools and especially those with the longest generation times are few generations removed from their wild progenitors.^13,32–34^ Because of these features, large-effect QTLs still segregate in breeding families, as evidenced by the large proportions of phenotypic variance explained by many QTLs detected in studies of cultivar×cultivar families. Rosaceae crop breeding germplasm is often highly heterozygous.^28,29^ An exception is peach; being self-fertile, breeders exploit selfing through multiple generations to fix desirable phenotypes.^15^ Rosaceae crop breeding germplasm typically has many alleles (or haplotypes) available for any given locus, even where heterozygosity is low.^35–41^ Bottlenecks due to radiation from a few founders reduces available alleles via largely unintentional inbreeding, such as for sweet cherry^42^ and peach to a lesser extent.^37^ Although just a handful of cultivars appear in the ancestry of most modern apple cultivars,^15^ there are still many alleles per locus because many and diverse other ancestors are also contributors to the cultivated crop^43,44^ and cultivar pedigrees.^45^

### Some further biological considerations

A long juvenility period lasting three or more years hampers breeding for most tree fruit crops of the Rosaceae family^46^ because breeders must wait this period to evaluate traits associated with flowers, nuts and fruit and to obtain gametes for creating the next generation. The use of rootstocks in commercial production of most Rosaceae tree crops can be considered an advantage to genetic improvement. Development of superior rootstock cultivars requires dedicated breeding programs. Such efforts are expensive and time-consuming, but less so than trying to assemble superior scion and rootstock attributes into single cultivars. For example, assembling the genetic factors underlying 10 attributes, each present at 50% frequency in base germplasm, into a single individual in two separate programs (2×0.5^10^) is 2000 times easier than assembling 20 such attributes into a new cultivar (0.5^20^); or 67 million times for 50 attributes. The existence of rootstocks complicates improvement where there are significant rootstock×scion×environment×management (for example, training system) effects. If a dwarfing- and precocity-inducing rootstock cultivar only imparts its desirable attributes to some scion cultivars under some growing conditions but not others, expanded trialing of combinations is warranted prior to widespread commercial deployment.

## THE PROMISE OF DNA INFORMATION FOR ROSACEOUS CROP BREEDING

### DNA information

Use of DNA-based information is compelling to support many areas of rosaceous crop breeding. Plant breeding constantly reinvents itself by integrating the latest innovations in science and technology.^47,48^ One such innovation with potential for enhancing many aspects of Rosaceae genetic improvement is marker-assisted breeding. This general concept of using information derived directly from the ‘genetic blueprint’ of crop plants for breeding purposes has been available for several decades, using genetic markers. Genetic markers are locus-specific tags that reveal polymorphism in the DNA sequence among individuals; DNA-based markers are the type most popular compared to earlier types (morphological, isozyme) especially because their abundance and ease of scoring.^30,49^ What comes to mind for most breeders and researchers when considering ‘marker-assisted breeding’ is performing selection among young seedlings, known as marker-assisted seedling selection.^16,49^ However, use of DNA-based genetic information (‘DNA information’ for short) has many other potential applications for rosaceous crop breeding.

### Characterization vs evaluation

Most applications can be classified into characterization and evaluation. Characterization is the determination of genetic organization, identity and relatedness of germplasm. Ascertaining unique identity, verifying and deducing paternity/parentage, pedigree and distant ancestry, as well as elucidating the structure of genetic diversity in a crop’s gene pools or specific germplasm sets are examples. Characterization is conducted with DNA markers that are neutral (that is, not necessarily associated with trait loci) typically assembled in ‘fingerprinting’ sets that can be just a few well-chosen multi-allelic markers (for example, Bassil *et al*.^50^) to genome-wide arrays (for example, Micheletti *et al*.^40^). These applications can help identify new breeding opportunities, avoid costly mistakes and streamline operations. Evaluation is the revelation of genetic potential for trait-based performance, which can be conducted with locus-specific DNA tests that are based on statistically significant, large-effect QTLs (for example, Longhi *et al*.^51^ and Sandefur *et al*.^52^) or with genome-wide DNA profiles that also capture small-effect loci for genome-wide selection (for example, Kumar *et al*.^53^). These applications can provide accurate assessments of genetic potential, enable efficient selection, reduce the number of generations during pre-breeding and shave years off trialing of elite selections. Both characterization and evaluation can be conducted by phenotyping, but the use of DNA markers via genotyping gives a direct window onto the many individual underlying genetic units that vary in breeding germplasm.

### Many breeding applications

Use of DNA information for characterization and evaluation purposes therefore has the potential to assist breeding activities and decisions to be more efficient, accurate, creative and rapid than using only traditional forms of breeding information such as phenotype and pedigree.^16,54^ Numerous practical applications of DNA information have already been undertaken in Rosaceae breeding and further applications beckon (Table 1).

### DNA-informed breeding

‘DNA-informed breeding’ encompasses the applications described in Table 1, and the term is defined here as the use of DNA-based genetic information, obtained via direct assays of an organism’s DNA, to directly support breeding decisions. Specific advantages of this term over ‘marker-assisted breeding’ is that it avoids the need to define markers and includes any use of DNA-based genetic information in breeding, beyond only ‘fingerprinting’ and QTL-targeted trait prognostics, such as genomic selection/genome-wide selection. Further, despite including the often-misinterpreted word ‘DNA’, in the author’s experience the term is readily understood by the layperson as an approach that monitors the genetics rather than adding, suppressing or editing genes of breeding germplasm. Therefore, DNA-informed breeding is unlikely to be misconstrued as being synonymous with genetic engineering.^83^ Yet, DNA-informed breeding can integrate with genetic engineering^48^ and gene editing where the latter technologies are conducted within a breeding context and DNA-based evaluation is used to detect the presence of inserted genes. DNA-informed breeding is therefore a versatile, multi-purpose approach that will surely endure in crop breeding for the foreseeable future.

### Genomics resources for DNA-informed breeding

Critical genomics resources in Rosaceae crops have been developed that lay the foundation for DNA-informed breeding.^1,7,15,84^ Most marker systems devised for plants have been adapted to Rosaceae and many have been used for developing fingerprinting sets (for example, refs,^50^ ^85–89^), genetic maps for a multitude of specific parents as well as crop reference maps (for example, refs 90–95), genome scans based on simple sequence repeat markers (SSRs; for example, Aranzana *et al*.^96^ and Silfverberg-Dilworth *et al*.^97^) and single-nucleotide polymorphism markers (SNPs; for example, refs 69 98–103). Also, well advanced in this crop family are ‘physical’ and physiological genomics resources^7,16,104,105^ including whole-genome sequences.^38,39,106–108^ Dozens of Mendelian trait loci (MTLs) and thousands of QTLs, which are genomic regions associated with statistically significant differences among individuals in qualitative and quantitative phenotypes, respectively, have been discovered and described (for example, Salazar *et al*.^6^ and Zorrilla-Fontanesi *et al*.^109^), and are compiled in the Genome Database for Rosaceae with the addition of research-enabling tools.^110^ Genomics resource development intended for breeding benefit in Rosaceae has been a necessary although underestimated undertaking, as described for apple.^2^ The diverse and promising applications of DNA information (Table 1) combined with the accumulation of vast genomics resources to support them represent great promise for revolutionizing breeding effectiveness in Rosaceae.

### The chasm

Promises of the genomics era for Rosaceae breeding have been fulfilled very slowly.^15,16,31,111^ Genomic resource development in this crop family diverted attention and resources from investments in traditional breeding capacity, as concluded for other crops.^48,112,113^ Yet most traditional operations such as acquiring germplasm, crossing, raising seedlings, trialing elite material and phenotypic evaluation have remained the backbone of new rosaceous cultivar development to meet industry and consumer demands, even as new molecular genetics tools became available.^1,1,13,29^ A disconnect was recognized between promise and practice, which in the US was termed ‘The Chasm’.^30,114^ Good intentions by genomics researchers were met by skeptical dismissal by breeders, such that genomicists continued conducting activities they were trained in and so did breeders, and the two were rarely united. In some cases, genomics research had been conducted for intended impacts in understanding Rosaceae crop physiology, and so lack of impact of that research on applied genetics (biodiversity management, breeding or cultivar choice by growers) can be assigned to the misconception that any study involving DNA is genetics. In other cases, the chasm appeared to widen because of the lack of formal or on-the-job training in translating genomics research outputs into breeding inputs, perhaps because of few success stories and their limited conditions, especially for clonally propagated, perennial crops. In contrast, success stories in genetic mapping, QTL discovery and genome sequencing mounted. Collard and Mackill^113^ called this the ‘application gap’—emphasis of scientists on conducting innovative research and its publication versus seeing the outputs through to practical application in breeding. To date, almost every scientific publication on rosaceous crops with ‘marker-assisted breeding’ or equivalent terms in the title describes a study that is upstream of actual application—only the promise. Nearly ten years ago, Moose and Mumm^48^ described for agricultural crops in general a growing need for the tide to turn, for genomics advances to be applied to breeding and the emphasis in genetic improvement to be shifted back to breeding itself. The dearth of practical use of DNA information based on trait loci until recently brings to mind that plant breeding should be wary of new technology bandwagons.^115,116^

### Bridging the chasm

A major shift to focusing on translation of genomics resources to practical breeding application is underway in Rosaceae, supported by large-scale projects. To bridge the chasm between genomics and breeding, focused attention on translational steps has been required.^31,117,118^ The large-scale, Rosaceae-wide, US-based RosBREED project (‘RosBREED: Enabling marker-assisted breeding in Rosaceae’, 2009–2014) galvanized scientists and stakeholders around this very premise,^31^ and is now in its second incarnation (‘RosBREED: Combining disease resistance with horticultural quality in new rosaceous cultivars’, 2014–2019).^119^ The European Union-based FruitBreedomics project, 2011–2015, targeting apple and peach also focused on ‘bridging the gap’.^15,120^ Stated one geneticist: ‘I assumed that discovering a QTL is all it takes to then be able to conduct marker-assisted breeding (MAB) … I started realizing that discovering a QTL is just the first step towards MAB … I have learned to appreciate the amount of work after QTL discovery that is required to develop a genetic test that can be used for routine MAB applications … Perhaps the issue is that not many researchers take the initiative to carry QTL discoveries all the way through to actual MAB to deliver impacts at the breeding end. Perhaps there are many researchers out there who still assume that QTL discovery is MAB’.^121^

## TRANSLATIONAL STEPS TAKEN

**Figure 1:**
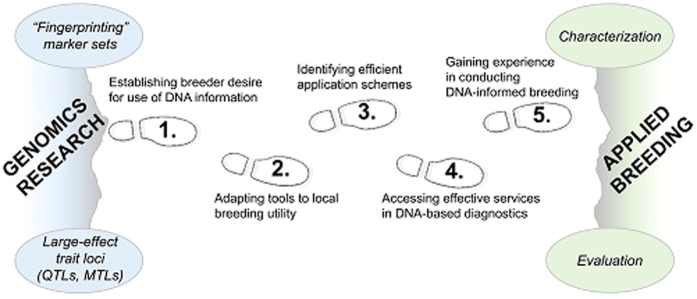
Five translational steps between outputs of genomics research and application of arising tools and knowledge applied routinely in breeding programs. Genomics and breeding are depicted as either end of a ‘chasm’ that is traversed by a focus on systematic translation. Fingerprinting sets of trait-neutral markers are converted for use in genetic characterization (identity/relatedness) of breeding germplasm. Knowledge of quantitative and Mendelian trait loci (quantitative trait locui (QTLs) and Mendelian trait loci (MTLs), respectively) is converted for breeding use in genetic evaluation (performance prediction), which includes genome-wide selection approaches accounting for these large-effect trait loci.

Successes in routine DNA-informed breeding for rosaceous crops have arisen when discoveries have been translated into practical breeding-friendly tools and knowledge. This translational path has been called the ‘MAB Pipeline’.^31,72,117^ The steps are conducted within breeding programs in a manner that fits each program’s idiosyncrasies and operates within, or provides the means to overcome, each crop’s breeding-limiting features (described earlier). In each case, the path begins with breeder pull and culminates with breeder experience. As expected, obstacles are frequently encountered, and many efforts fall short at one step or another prior to delivering breeding impact. Pitfalls hinder progress and appear only as they are approached or usually too late (such as a sample labeling error detected after genotyping), for which solutions must be devised at the time to get back on track (such as re-sampling as well as adding an extra layer of quality control to prevent future errors).^122^ Limitations suppress the downstream positive impact of a well-executed approach (such as a lack of large-effect allelic contrasts in the program’s germplasm for a high-priority trait) and require large shifts in the approach to change the status quo (such as infusion of new germplasm into the program or consideration of a genome-wide selection rather than QTL-targeting selection approach for that trait). Most importantly, the stepwise approach engenders systematic consideration and effort rather than *ad hoc* hope and serendipity in reaching success. Below, five key steps (Figure 1) and reported examples are described. Their systematic consideration is expected to help ensure the future flow of benefits from outcomes of the genomics era into genetic crop improvement.

### Step 1: Establishing breeder desire for use of DNA information

A ‘pull’ from breeding for the possibilities and outputs of genomics research occurs when breeders understand and request translation of generic tools for use in addressing their program’s needs. This understanding requires awareness of the state-of-the-art—DNA-informed breeding technologies, strategies and experiences of others—for their crop. Public institution breeders typically remain current by reading primary literature boosted by research of their students and postdocs; this avenue is open but less expected for private breeders. The FruitBreedomics project held annual ‘Stakeholder days’ (where stakeholders were breeders, genebank curators and industry representatives) during the project’s four-and-a-half years to engage European peach and apple breeders in DNA-informed breeding advances.^15,123^ Similarly, in mid-2012 and mid-2013 the RosBREED project held general breeder workshops,^121,124,125^ with crop-specific workshops in between.^126–130^ RosBREED also conducts in-person breeder visits^131–133^ to raise and maintain awareness of DNA-informed breeding opportunities, as well as disseminating an ongoing ‘Community Breeders’ Page’ column targeting the US and wider community of rosaceous crop breeders with articles focused on upstream research approaches, technology interfacing, new DNA information and events.^134^ Both FruitBreedomics and RosBREED had/have websites with breeder-oriented resources; in RosBREED, this is found on the ‘For Breeders’ section (www.rosbreed.org/breeding) of the main project website.

### Step 2: Adapting tools to local breeding utility

Knowledge of trait locus genomic positions, the output of most QTL studies, must be converted into locus-specific, performance-predictive assays that are relevant to alleles segregating in a program’s germplasm and amenable to available DNA-based diagnostics services. Validation of the breeding significance of QTLs is often conducted by examining a set of cultivars; Peace *et al.*^45^ argued against relying on such germplasm because of selection bias, and instead demonstrated a strategy that places attention on the average allelic representation by unselected offspring of a breeding program’s important breeding parents. Rather than assuming no family structure among a set of cultivars or relying on the limited number of alleles segregating in and genetic background of a single mapping family, the strategy exploits Pedigree-Based Analysis^135,136^ to determine statistical significance for locus effects on phenotype across multiple, various-sized, pedigree-connected families, which exemplifies breeding germplasm.

After a breeder is confident that a trait locus is relevant for their program’s germplasm, a ‘DNA test’ targeting the locus that at least differentiates the high-value allelic contrasts in genetic potential associated with the original QTL is needed. Such locus-specific DNA tests might be developed in centralized labs separate from breeding programs, but are suitable only if the DNA marker type or platform being developed suits a breeding program’s service provider (step 4) and price point (step 3). Once a candidate DNA test is developed for a trait locus, the systematic QTL validation strategy^45^ can be used to confirm phenotype–genotype associations. In the case of MTLs, a subset of individuals representing alleles of interest can be sufficient to ensure the DNA test differentiates alleles and genotypes expected to exist in the breeding program.^52,137^

To optimally deploy a DNA test, breeders must know the effects, sources, frequencies and distributions of alleles revealed in their program’s germplasm. Sandefur *et al.*^52^ reported a DNA test for cherry fruit color that described these utility components for a particular cherry breeding program. Such a test could be adapted to another program by repeating some germplasm individuals as standards for certain alleles and adding important breeding parents of the second program. Sets of individuals with phenotypic contrasts of interest should also be included for confirming effects of alleles revealed by the DNA test.

Not all conversions of QTLs into DNA tests, or transfers of DNA tests developed for one program to another, are successful. Technical hurdles include markers not revealing sufficient polymorphism or having genotypes that are difficult to distinguish. Additional candidate assays can be trialed to overcome such problems. Biological hurdles include the DNA test not explaining as much phenotypic variation as reported for the QTL/other program or the desired phenotypic prediction not being evident for some or all individuals (for example, refs 137–139). In the former situation, the germplasm level at which the DNA test is to be routinely applied can be changed, usually from seedlings, for which a terminal decision is involved, to parents and elite selections, for which the DNA test’s information can be weighed with much else. In the latter situation, a new closer marker might overcome recombination or those lineages with a different linkage phase of marker and QTL alleles could be monitored separately. Further troubleshooting could involve testing the hypotheses of a similar phenotype resulting from an alternative mutation at the same locus (for example, ‘my yellow flesh allele is not the same as yours’)^140^ or a different locus (for example, ‘my source of blood flesh is not the same as yours’).^141^

A systematic compilation of information about DNA tests is being implemented in the RosBREED project: ‘DNA test cards’. These double-sided handouts present key information about each test in a consistent format, are delivered to breeder ‘clients’ (any US Rosaceae crop breeder), and can be updated regularly as allele effects are refined, new alleles are discovered, additional loci are included or marker types are changed.^142,143^ Information is also provided online,^144^ which allows room to list the allelic combinations for publicly available cultivars and ancestors revealed by some DNA tests.^145^ However, preparation of DNA test cards is not simple and cards are not keeping pace with the many reported DNA tests—for example, Evans and Peace^2^ list ~40 DNA tests spanning 16 traits currently available for apple, but only one of these is currently in DNA test card format.

Simple PCR-based markers such as SSRs and sequence-characterized amplified regions have been used most commonly in DNA tests developed for seedling screening to date, likely for several reasons. They are amenable to many genotyping platforms, robust to a wide range of DNA extract qualities and quantities, versatile for use on subsets of a larger germplasm set such as specific families segregating for only some loci or specific seedlings to be kept following another test, and can be combined (multiplexed) readily with other DNA tests.^146^ SNP-based DNA tests are gaining popularity (for example, refs 147–149), and machines and platforms on which they can be efficiently run are increasing. Versatility and cost considerations are most relevant for seedling families in MASS and MAI (Table 1); evaluation of elite germplasm often involves all available DNA tests but the low number and high value of germplasm relieves cost concerns.^146^

Sets of DNA markers for fingerprinting must also be relevant to local germplasm and diagnostics services. Multiplexed sets of 10 to twenty simple PCR markers such as SSRs^41,50,60,150^ provide low-cost and informative assays for many breeding germplasm characterization applications (Table 1). SNP arrays (with tens to thousands of SNPs) are expected to prove increasingly useful for fingerprinting applications. However, as for trait locus-targeting DNA tests, fingerprinting sets should be confirmed for effectiveness on breeding germplasm prior to widespread use.

### Step 3: Identifying efficient application schemes

The inherent expense and time involved in developing new rosaceous cultivars necessitates improvements in efficiency. With a wide range of individual breeding operational situations, costs, and predictiveness of DNA tests, decision support to help breeders identify resource-efficient application schemes would be useful.^49^ Modeling fruit breeding that included the rosaceous crops of apple and strawberry, Luby and Shaw^151^ recommended some general conditions under which MASS should be cost-efficient. This modeling was extended by Edge-Garza *et al.*^152^ to model costs of breeding operations over time, allowing for normal losses of seedlings during various stage of seedling-raising operations. A Microsoft Excel spreadsheet-based ‘MASS Efficiency Calculator’ was provided that can dissect complex breeding scenarios, enabling breeders to explore the cost efficiency of possible MASS schemes relevant to their own situation. Cost-efficient situations were determined to be common in breeding programs typified by most rosaceous crops, in which large costs are traditionally incurred by growing and evaluating plants in outdoor field for several years. The software was reprogrammed in Python language with additional features^153^ and made available online.^154^

Genetic gain is another efficiency consideration. Cost-efficiency calculations described above assume that seedlings discarded because of inferior DNA test genotypes would have similarly been discarded by phenotype under traditional seedling selection (TSS), which is unlikely unless both broad-sense heritability (*H*) and the proportion of that heritability explained by the DNA test, the broad-sense predictiveness (*P*) are both high. Ru *et al.*^14^ conducted computer simulations of genetic gain efficiency of several forms of MASS (marker-only, two-stage and index) compared to TSS across a wide range of *H* and *P* values for single traits, from which a decision-support framework was developed. The key to optimal deployment of MASS was concluded to rely on the ratio of *P* to *H*. Where *P*>*H*, that is, the DNA test captures most of the genetic effects, only the best genotypic class of seedlings should be kept and thus the DNA test is relied upon heavily. A DNA test detecting presence/absence of a high-penetrance resistance allele is an example of this case. Where *P*<*H*, only the worst seedling should be discarded, thus reliance on the DNA test is minimal, enabling other assays for genetic potential such as phenotype, further DNA tests or GS to try to capture the remaining genetic effects prior to a keep/kill decision. DNA tests for QTLs (as opposed to MTLs) often fall into these *P*<*H* cases, mirroring the intuition of many breeder in deploying such tests; however, where heritability is low yet a DNA test captures most of the genet effects (an example would be a DNA test based on the fruit fructose content QTL on linkage group 1 of apple),^155^ the ‘rely upon heavily’ deployment strategy of MASS is most efficient both in terms of genetic gain and cost. The model of Ru *et al.*^14^ is being validated empirically and integrated with cost considerations.

Because DNA-informed breeding has many more applications than just MASS (Table 1), the RosBREED project is currently developing a wider framework to identify compellingly efficient uses of DNA information across breeding operations. While fast-paced, high-tech, high-throughput MASS tends to garner most attention, and using trait-predictive DNA tests at other germplasm levels is also attractive, the highest benefit: cost ratio is likely to come from germplasm characterization for most breeding programs. Given how often DNA information reveals that records and plants are not what breeders and growers believed (for example, refs 13,27,58,61,95), applications such as ensuring introduced parents are true-to-type, checking crossing success, confirming and deducing parentage, ensuring the intended selection is planted and ensuring the correct new cultivar is mass-propagated are very compelling.^133^ In these cases, costs of DNA fingerprinting are relatively low whereas proceeding on false knowledge hampers breeding progress ^27^ or can even be disastrous.

### Step 4: Accessing effective services in DNA-based diagnostics

With breeding-relevant DNA tests or fingerprinting sets available and efficient deployment schemes identified, breeding programs need access to DNA-based diagnostics services to obtain the DNA information on their germplasm. Services must be cost-effective and timely, with streamlined tissue-sampling operations. Cost structures of commercial or research-subsidized services can be incorporated into cost-modeling software such as that described above. In MASS that involves thousands of samples, time taken to conduct tissue sampling, DNA extraction, genotyping and provision of results back to the breeder is a major logistical consideration. Time taken from sampling through to killing can be compared to the windows of opportunity during breeding operations to raise and handle seedlings. Some windows are only a few weeks in duration, such as from when most seedlings reaching a minimum number of leaves in the greenhouse until they need to be moved to a new location or pot size. Edge-Garza *et al.*^152^ incorporated these logistical considerations into cost-modeling, and suggested that when time constraints exist DNA testing can be spread across multiple cost-efficient stages, especially testing as many seedlings as time allows in the most cost-efficient stage. In any case, decision-support tools to estimate relative savings achievable from possible testing stages enables quantitative consideration of the resource-efficiency consequences of a breeding program’s operational set-up.^52^ On the service-provider side, time constraints might be alleviated by spreading the workload over multiple technicians working simultaneously.^152^ Ease of tissue sampling for high-throughput MASS in rosaceous tree fruit crops was the primary driver for the adoption of the silica bead method of DNA extraction, which avoids the need for tissue samples to be large, individually labeled, kept cold, freeze-dried or laboriously ground.^156^ Using the silica bead method, thousands of seedlings can be readily sampled and DNA-extracted in a week cheaply and by breeding personnel rather than specialists. Automation is another means of easing logistical constraints as well as reducing error through less human handling, such as the commercial service now routinely used for MASS in a rootstock breeding program.^157^

A key breeding need from DNA-based diagnostics services is readily interpreted results—certainly not raw electropherograms or SNP calls. Presence/absence tests for MTLs such as for a particular source of disease resistance or *S*-genotypes of new cultivars are relatively simple; multi-locus QTL evaluation less so. For MASS needs of the Washington State University apple breeding program, my ‘service’ (research) laboratory developed an 8×12-sample ‘Keep/Kill’ color-coded result for every set of 96 seedlings which includes a qualitative summation of all genotypes detected for each seedling.^2,72^ With this approach, individual decisions underlying the terminal result can be viewed and adjusted by the breeder in real time. However, a time-efficient approach is to establish selection decisions and contingencies prior to receiving test results and then view results in real time only to monitor progress.

### Step 5: Gaining experience in conducting DNA-informed breeding

When breeders obtain hands-on experience with integrating DNA information into routine breeding operations and decisions, they can identify pitfalls and devise solutions, recognize limitations and adjust expectations, and enjoy successes in breeding efficiency, accuracy, creativity and speed toward superior new cultivar releases. Each crop’s plant-based features (described earlier) that frame traditional breeding also greatly affect the integration of DNA information, the particulars of which are compounded with each program’s idiosyncrasies in traits of interest, germplasm used, available resources, budget and personalities of the breeder and staff. Yet challenges and opportunities are rarely entirely idiosyncratic—by sharing experiences, programs can adopt successful strategies and anticipate problems. These experiences are sometimes reported (for example, in Europe^74,123,158^ and the US^2,62,68,72,159,160^) but participation in professional society conferences and collaboration are the usual means of learning from others in this fast-paced field.

Following are examples of experiences in DNA-informed breeding in the Washington State University apple breeding program that led to course corrections. MAPS has proven more efficient than MASS: crosses made to avoid the worst allelic combinations according to two DNA tests used in 2008 on parents ^2,161^ had eliminated the need for one of the DNA tests in MASS by 2012 and the other has only been used sporadically since routine MASS began in 2010. Much attention therefore focuses on MAPS. In conducting MASS, although the greenhouse stage was determined to not be as cost-efficient stage as a later nursery stage,^72^ former seedlings were better labeled and easier to access, leading to fewer opportunities for errors in genotype-plant matching, and the relative savings were only marginally less.^152^ Therefore, the greenhouse stage became the standard for MASS operations.^2^ A custom-designed 8×12 seedling pot format was established to streamline the killing procedure by easy matching of DNA test results to greenhouse plants, reducing errors.^2,162^ The adjusted pot system also enables sorting within families by growth rate prior to tissue sampling and eases removal of seedlings for various reasons and consolidation of those remaining. Each set of 96 has a single label in the greenhouse and DNA testing lab, and positive and negative controls are included in the same position in each set of 96 to enhance quality control in the lab.^2,122^ DNA testing of thousands of seedlings for trait loci has simultaneously detected seedlings resulting from unintended paternal parents; crossing operations were adjusted several years ago when the proportion was deemed too high.^2^ In this program, genetic evaluation has also enabled genetic characterization for elite selections: genotypic data from routine DNA testing of selections at trait loci of interest were quickly co-opted for verification or deduction of parentage records.^2^

## IMPACTS OF DNA-INFORMED BREEDING IN ROSACEAE

### Lack of documentation

Use of DNA information is becoming conventional in rosaceous crop breeding, contributing to many decisions and operations, but impacts have not yet been scientifically documented. While DNA information has been used in numerous rosaceous crop breeding programs, since the first germplasm relatedness findings were considered in a breeding context around the 1990s and the first valuable alleles were tracked around the turn of the millennium, the impacts of use have received little attention in scientific literature. This lack of documentation is not surprising, given that descriptions of application and what happens subsequently are not suitable for most scientific publications especially those with high impact factors that public research institutions encourage their scientists to target, and given the little disclosure from private institutions.^113^ Yet, documented impacts would help justify both the fundamental research and its translation to practice, and could be readily addressed as hypothesis-driven science.

### Documentation is possible

Reported ‘deliverables’ of the RosBREED project^63,163,164^ were categorized as knowledge, tools and germplasm. The definition of knowledge deliverables of using DNA information included new breeding strategies, new protocols, information on plant identity, trait genetics, pedigree information and genetic potential of breeding germplasm, and experience using DNA markers in breeding programs. Tools were defined as ‘DNA tests and software’ (fingerprinting sets would also fit here). Germplasm deliverables were defined as ‘access to new gene pools, new parents, new progenies, promising selections and cultivar releases’. The impact of using DNA test tools to gain knowledge of parents—their performance-associated trait locus alleles—and thereby guide crossing decisions (MAPS; described for four US breeding programs)^163,164^ is surely great, infusing the next generation with superior alleles that should lead to additional or better released cultivars. How many more? How much better? Positive changes generated by DNA information use in quantifiably enhancing new cultivar development have thus far only been implied. Empirical validation of the impacts of DNA-informed breeding application (which is not validation of trait loci, markers, DNA tests or fingerprinting sets) is sorely needed in Rosaceae.

### Some quantified impacts

Some attempts have been made to quantify impacts of DNA-informed breeding, although less formally than as scientific experiments and with indirect evidence only. Reports in the RosBREED project’s periodical newsletter^63,163^ and in conference proceedings described breeding outcomes based on a comparison of what was done to the hypothetical situation of no DNA information used. Gains in cost-efficiency, usually described in the context of the estimated monetary value of resources saved (by avoiding further costs of raising and evaluating killed seedlings) and thereby able to be more effectively allocated in the program, were reported for several programs. An estimated savings of at least $160 K from MASS in 2010–2012 was reported for the Washington State University apple breeding program.^162^ In the same program, a further $82 K savings was estimated from MASS in 2013 and 2014 on 16 000 seedlings with 66% killed;^165^ over seven seasons of MASS in 2010–2016, ~56% of 45 000 screened seedlings have been killed.^2^ For the University of Minnesota apple breeding program, by killing half of more than 6000 seedlings screened in 2013 and 2014 an estimated $40 K in future costs was saved.^165^ MASS in sweet cherry breeding at Washington State University was estimated to provide resource savings of $75–80 K in 2010 and 2011 by killing more than half of almost 3000 seedlings tested.^8,68^ The following two years, 2013 and 2014, was associated with savings of more than $80 K by killing 85% of 3400 seedlings.^165^

### DNA-informed breeding is the new convention

DNA-informed breeding is now conventional for Rosaceae, in the US at least. Surveys in 2010 and 2014 of the US breeder community^166^ revealed that as of 2014 two-thirds of 40 responding breeders use DNA information for genetic characterization (42% in 2010), 50% for MAPS (39% in 2010) and 34% for MASS (32% in 2010), and that breeders increasingly associate genetic testing and markers with practical breeding program applications rather than upstream research.^118^ For peach and apple in Europe, DNA-informed breeding also appears to be the norm.^123^ Although only some germplasm and operations in such breeding programs is routinely DNA-tested and/or fingerprinted, the increasing adoption of this approach as conventional suggests that, all else being equal, breeders who aren’t DNA-informed will fall behind the competition.^123^ Current trajectories point toward more DNA tests for more traits and explaining more of the genetic variation in breeding germplasm, genetic characterization at greater genomic resolution, more streamlined and cheaper diagnostics services and continued collaboration among the worldwide Rosaceae genomics, genetics and breeding community to tackle larger problems and more of the details.

## NEXT LEAPS NEEDED

### Expanding to genome-wide considerations and software

DNA-based diagnostics is now expanding from specific loci to genome-wide considerations. Realizing the full potential of this expansion for more efficient, accurate and creative breeding advances will require research leaps in several areas. Accuracy of trait performance predictions from DNA information needs an overhaul. Phenotyping of diverse germplasm on which effects are estimated should become more physiologically informed, consumer-informed, objective, standardized, considerate of environmental interactions and hierarchical.^167,168^ Improved statistical models are needed that describe the effects of allelic combinations at specific trait loci for the target traits as well as all others of interest. By accounting for genetic background, non-genetic effects and genotype×environment×management interactions, predictions could be made of corollary effects of specific selection decisions,^169^ helping to improve breeder confidence in DNA information. Multi-trait DNA profiling information and tools are required to efficiently evaluate breeding germplasm for all alleles influencing all traits of interest. Although large-effect trait loci abound in rosaceous breeding germplasm, the many traits under selection consideration combined with often multiple loci per trait lead to the extreme likelihood that selection pressure on any DNA test-targeted locus will affect other traits. A genome-spanning set of informative markers for each crop, both locus-specific and genome-wide, could capture these major genetic effects. Combined with genetic characterization of ancestry and recombination, the information could be readily interpreted and creatively manipulated by breeders—effectively enabling ‘breeding by design’.^170,171^ RosBREED’s haploblocking approach targets such outcomes. At the right resolution, such DNA profiling could also efficiently capture the cumulative effects of many tiny, individually non-significant loci. Indeed, for genetic variation in breeding germplasm best explained by the additive and interactive effects of many tiny-effect alleles, the technique of GWS appears promising in Rosaceae especially in combination with QTL- and MTL-based selection.^53,65,76,172^

Breeding information management tools are desperately needed to handle the new types of DNA-based data increasingly available to breeders.^110,173^ Software is the solution, to provide new tools that combine breeders’ ideas and targets with DNA profiles and performance data. The better the software, the more effectively it should enable breeders to access all information that is available about their germplasm, especially to enhance creativity by encouraging the question ‘’What would I get if I crossed…?’^174^

### Overcoming plant-based limitations

Targeted strategies are needed to overcome plant-based biological features currently limiting breeding progress. Some are obvious applications of existing tools and approaches. Dissecting trait genetic complexity can help breeders prioritize suitable targets. Some crossing challenges can be overcome by revealing cross-compatibility alleles and verifying the effectiveness of alternative crossing methods. Application of robust DNA tests can ease the selection process. In the presence of high G×E, alleles can be sought for desirable attributes that provide phenotypic stability across environmental conditions likely to be encountered in commercial settings. The existence of desirable genetic diversity can be revealed by allele-mining with DNA tests for valuable traits. Low heterozygosity can be addressed by seeking highly heterozygous parents or those with specific alleles to encourage diverse outcomes in seedlings in desired trait directions. Certain attributes could be genetically investigated primarily to serve ‘selfish’ breeding needs: short juvenility, small plant size and ease of propagation. DNA-informed breeding can also be integrated with other innovative strategies. Rapid generation cycling techniques,^46^ including the intermediately-transgenic early flowering method,^175^ have the potential to mitigate the key breeding hurdle of long juvenility especially when combined with MAI^13^ (Table 1). Where there is a lack of allelic variation for critical traits, DNA information can help with germplasm diagnostics for new technologies that access the quaternary gene pool (genetic engineering) or create new alleles (gene editing).

### Training in translational genetics

Finally, training of current and future breeding personnel and allied scientists is needed in this relatively new field. Translational activities are new and not routinely taught, but require many expert practitioners among breeding personnel and allied scientists for widespread delivery of genomics benefits to Rosaceae breeding. In addition to existing personnel, the next generation of professionals in horticultural breeding and genetics need skills, knowledge and experiences in DNA-informed breeding. The RosBREED project has directly involved several dozen breeding programs with their breeders and staff,^31,176^ who have gained much experience with understanding and using DNA information; the FruitBreedomics project similarly engaged many on-the-job breeding personnel. RosBREED also trained many graduate students (31 graduated at last count^177^)—which perhaps will be the most far-reaching impact of the project.^178–180^

Formal plant breeding education needs changing. Some publications have recommended that modern plant breeding education should include molecular biology, such as knowledge of gene function and experience with laboratory methods of molecular biology and functional genomics.^48,181^ While such training familiarizes students with both sides of the ‘chasm’, it does not in itself address its bridging. Integration is restricted when breeders and molecular biologists do not understand each other’s concepts and jargon,^113^ but I contend that current and future breeders do not need to be molecular biology experts, and vice versa for molecular biologists. Instead, as emphasized by Baenzinger^182^ and Repinski *et al.,*^183^ successful plant breeding occurs with multidisciplinary teams. Breeding is the central hub but there is division of expertise and labor. Understanding how to integrate molecular genetics with traditional breeding is expected of both private and public plant breeders^181^—integration is key, not the individual components. An alternative approach for instilling expertise in current and future professionals is to consider translational concepts and practices as its own specialty. This specialty lies in between breeding and molecular genetics/genomics (and not ‘molecular biology’, which is either too broad to be directly relevant to new cultivar development or is a field within the discipline of physiology). In this scheme, a professional would focus their expertise and efforts in one specialty—breeding, translational genetics or molecular genetics/genomics—but be familiar with the concepts and jargon of the adjacent specialty(s). Perhaps the biggest challenge for new PhD training will be the focus of research-based chapters of the dissertation—getting out of the mindset that these should be about QTL discovery or similar; instead, research can focus on translational concepts and application hypotheses.

## CONCLUSION

Breeding of rosaceous crops has entered a new era—in practice, not just promise. Use of DNA information to support at least some routine breeding decisions and operations is now conventional in the US and probably elsewhere, and is growing in programs and kinds of applications. The more systematic, frequent and routine this DNA information use in a breeding program, the greater the benefits flowing from fundamental advances in understanding genetic variation, inheritance, genomic organization and phenotypic performance into developing superior new cultivars. Challenges remain in translational genetics, but experiences and successes by a growing number of practitioners lights the way. DNA-informed breeding can increase operational efficiency by reducing costs, time and other limited resources. By revealing genetic potential at the level of DNA sequence variation, DNA-informed breeding improves accuracy over traditional phenotypic evaluation. DNA-informed breeding also places breeders in the creative driver’s seat. With many DNA-based tools and knowledge about their crop’s and program’s germplasm, breeders have an abundance of possibilities at their fingertips. The simple basis of this approach—monitoring genetic variation directly—and its versatile applications render it free from ties to specific technological platforms; DNA-informed breeding appears as relevant to the future of rosaceous crop breeding as breeding is to the future of crop improvement.

## Figures and Tables

**Table 1:** Some possible DNA-informed breeding applications for rosaceous crops

*Stage*	*Type*	*Application*	*Example(s) reported in Rosaceae*
Goal-setting	Eval	Better understand trait genetics to define breeding targets or strategies	Apple^55–57^
			
New genetic variation	Char	Identify genomic regions under selection, including segregation distortions, to target or improve segregation predictions	
	Char	Determine parentage of cultivars and elite selections to confirm or refute assumptions about trait inheritance	Apple;^58,59^ Pear^60^
	Eval	Identify valuable alleles beyond the program’s germplasm to find new parents (allele mining)	
	Char	Determine identity of parents to avoid growing and using wrong individuals	Apple^61^
	Char	Calculate relatedness in the parental gene pool to identify opportunities for infusing new alleles (MAPS)	Apple^62^
	Eval	Identify valuable alleles in pre-breeding seedlings to choose suitable new parents during introgression (MAI)	Sweet cherry^63^
	Char	Identify pre-breeding seedlings with most suitable genomic backgrounds (MAI)	Almond^64^
	Char	Calculate relatedness among parents to identify crosses to avoid (MAPS)	
	Eval	Identify valuable genome-wide profiles among parents to identify best parents for contributing superior attributes (GWS/GS)	Strawberry^65^
	Eval	Identify valuable alleles in the parent pool to choose useful parent combinations (MAPS)	Apple;^2,66^ Cherry^67,68^; Strawberry^4^
	Char	Determine parentage of seedlings to evaluate crossing method success	Apple;^2^ Rose;^69^ Sweet cherry^70^
			
Selection	Char	Determine parentage of seedlings to kill those without desired parentage	
	Char	Determine parentage of seedlings to identify labeling errors	Sweet cherry^70^
	Eval	Identify valuable alleles in seedlings to kill those predicted to be genetically inferior (MASS)	Apple;^2,66,71–73^ Peach;^74^ Sweet cherry^68,70,75^; Strawberry^4^
	Eval	Identify valuable genome-wide profiles among seedlings to kill those predicted to be genetically inferior (GWS/GS)	Apple^76^
	Eval	Identify valuable alleles in seedlings to sort into categories for differential evaluation	
	Eval	Identify seedlings successfully pyramided for multiple resistance alleles for a disease	Apple^73,77^
	Eval	Identify valuable alleles in elite selections to support advance/discard decisions	Cherry^68^
	Eval	Identify valuable alleles in elite selections to determine suitable trial conditions	
			
Commercialization	Eval	Identify valuable alleles in cultivar releases to supplement performance descriptions	Apple^78–80^
	Eval	Identify valuable alleles in cultivar releases to provide insight on management options	Various^81^
	Char	Determine parentage of cultivars and elite selections to support patent applications	Apple;^78^ Strawberry^82^
	Char	Assign unique identity to cultivars to support patent applications and deter theft	Apple^78^; Strawberry^4^
	Char	Determine identity of elite selections and cultivar releases to avoid or detect errors in clonal propagation	Apple^2,62^; Strawberry^4^

Abbreviations: Char, characterization (identity/relatedness applications); Eval, evaluation (performance prediction applications); GS, genomic selection; GWS, genome-wide selection; MA, marker assisted; MAI, marker-assisted introgression;^64^ MAPS, marker-assisted parent selection;^30^^,^^49^ MASS, marker-assisted seedling selection.^2,30,^^49^
